# Performance Score (T2D)—A New Perspective in the Assessment of Six-Minute Walking Tests in Pulmonary Rehabilitation

**DOI:** 10.3390/diagnostics12102402

**Published:** 2022-10-03

**Authors:** Barbara Wagner, Andrej Zdravkovic, Michael Pirchl, Milo A. Puhan, Ralf Harun Zwick, Vincent Grote, Richard Crevenna, Michael J. Fischer

**Affiliations:** 1 Department of Physical Medicine, Rehabilitation and Occupational Medicine, Medical University of Vienna, Waehringer Guertel 18-20, 1090 Vienna, Austria; 2 Ludwig Boltzmann Institute for Rehabilitation Research, Kurbadstrasse 14, 1100 Vienna, Austria; 3 Epidemiology, Biostatistics and Prevention Institute, University of Zürich, Hirschengraben 84, 8001 Zürich, Switzerland; 4 Therme Wien Med, Kurbadstrasse 14, 1100 Vienna, Austria; 5 Vamed Rehabilitation Center Kitzbühel, Hornweg 32, 6370 Kitzbühel, Austria; 6 Hannover Medical School MHH, Clinic for Rehabilitation Medicine, Carl-Neuberg-Straße 1, 30625 Hannover, Germany

**Keywords:** COPD, six-minute walking test, exercise capacity, pulmonary rehabilitation, outcome measure

## Abstract

Because absolute changes in outcomes are difficult to interpret and the minimal clinically important difference (MCID) is not suitable to address this challenge, a novel method of classifying outcomes by relating changes to baseline values is warranted. We used the “performance score” (T2D), which reflects individual performance, enabling us to consider the functional status at the beginning of rehabilitation without dealing with the problems of mathematical coupling or regression effects, as encountered in ANCOVA. To illustrate the T2D, we retrospectively analyzed changes in the six-minute walking test (6MWT) in COPD patients undergoing outpatient pulmonary rehabilitation and compared the results with absolute differences related to a predetermined MCID. We evaluated a total of 575 COPD patients with a mean age of 61.4 ± 9.2 years. 6MWT improved significantly, with a mean change of 32.3 ± 71.2. A total of 105/311 participants who had reached the MCID were still classified as “below average” by the T2D. Conversely, 76/264 patients who had not reached the MCID were classified as “above average”. This new performance measure accounts for the patient’s current status and for changes over time, potentially representing a simple and user-friendly tool that can be used to quantify a patient’s performance and response to rehabilitation.

## 1. Introduction

Chronic obstructive pulmonary disease (COPD) is a common disease and a leading cause of morbidity and mortality worldwide. The global prevalence of COPD is increasing [1], with current estimates ranging between 8 and 15% [2]. According to the World Health Organization (WHO), COPD was the fifth leading cause of death for both sexes in Austria in the year 2019 [3]. Chronic debilitating respiratory symptoms and acute exacerbations are hallmarks of the disease and significantly compromise patients’ health and quality of life [4,5]. COPD is associated with multiple comorbidities, as well as an increased risk of cardiovascular events and mortality; thus, it represents a considerable health and socioeconomic burden [5,6].

Aside pharmacological treatment, pulmonary rehabilitation, self-management education, and the treatment of comorbidities are important aspects of the holistic management of the disease [4]. Pulmonary rehabilitation is known to enhance health-related quality of life [7,8]. Exercise has beneficial effects on peripheral skeletal muscle strength and exercise capacity [9]. Resistance training improves maximal strength, as well as muscle endurance and power [10]. Aerobic exercise can effectively improve dyspnea, exercise capacity, and quality of life in patients with COPD [11,12]. (Outpatient) pulmonary rehabilitation and respiratory physiotherapy interventions have been shown to be cost-effective in terms of costs per unit of quality of life gained and quality-adjusted life years and can even save costs, a goal that is extremely difficult to attain with respect to medical treatment options [13,14]. 

Clinician-reported outcome measures are used in rehabilitation to assess and track the patient’s health status and convalescence. In practice, measuring any such improvements often proves difficult, as differing baseline health issues must be considered. Patients who show satisfactory initial results are expected to improve less throughout the course of treatment than those with poor baseline values. Therefore, a performance assessment that adequately reflects relative improvements while considering floor and ceiling effects is warranted. Establishing MCIDs for patients with differing baseline values is challenging, as distribution and anchor-based methods are sensitive to the distributions of the measure of interest or anchors when performed within patient strata and are likely to result in invalid estimates. 

A novel integrated performance measure was introduced in 2021 that addresses the challenge of considering a broad range of baseline values. The “performance score” (T2D) is a distribution-based approach that can be used to describe changes in outcomes over the course of treatment, thereby putting absolute values into perspective. [15,16]. In previous studies T2D was applied to inpatient rehabilitation in patients with low back pain and following traumatic injuries of the lower limbs [16,17]. In this study, our aim was to apply the T2D in the context of pulmonary rehabilitation, where much work on the MCID has been done. The exercise tolerance of COPD patients was investigated using the six-minute walking test (6MWT) [18,19]. The 6MWT is a valid assessment of functional exercise capacity and can be carried out across the clinical spectrum of the disease [20]. It is used frequently both in routine clinical practice and as a primary end point for research in patients with COPD [20,21]. The 6MWT can be used to assess the response to therapy, as it is sensitive to commonly used therapies in COPD, including pulmonary rehabilitation [20]. For patients with chronic respiratory disease, a minimal clinically important difference (MCID) of 30 m has been suggested [22]. The 6MWT is also used to assess prognosis and is considered to be a strong predictor of mortality [20]. 

In this study, we applied the T2D in COPD patients for the first time to evaluate changes in 6MWT and to differentiate between above- and below-average performance in patients undergoing outpatient pulmonary rehabilitation. 

## 2. Materials and Methods

### 2.1. Study Design

This is a retrospective, single-arm cohort study. 

### 2.2. Patient Collective

Data obtained from COPD patients who underwent phase II and phase III rehabilitation in an outpatient setting according to the medical service profile of outpatient rehabilitation for patients with pulmonary diseases [23] were considered for data analysis. Data were collected at a specialized Austrian pulmonary rehabilitation center between January 2012 and December 2020. Only patients who had undergone at least 75% of prescribed therapy sessions and had performed a 6MWT at baseline and at the end of rehabilitation were included. 

### 2.3. Intervention

Treatment in phase II rehabilitation consisted of 60 therapy sessions (including four “non-therapeutic” units, which involved medical examination and diagnostics) lasting 50 min each over six weeks. Patients usually received 3.0–3.5 (at least 2.5) therapy sessions a day and had at least three days of treatment a week. At least 80% of treatment units consisted of exercise therapy, physiotherapy, and respiratory therapy. At least six therapy sessions were performed in a one-on-one setting. Exercise therapy was individually adapted in terms of exercise intensity, type, and progression. Four to six therapy sessions focused on education concerning COPD and its treatment (including inhalation devices and home oxygen therapy), as well as advice for smokers, healthy nutrition, and lifestyle [23]. 

Patients in phase III outpatient rehabilitation had either 45, 67.5, or 90 treatment sessions approved by their insurance carrier (including 4.5 “non-therapeutic” units) for six, nine, or twelve months, respectively. In general, patients performed 1.5–2.5 treatment units (at least 1.5) at least two days per week. The 6MWT was performed once at baseline (t1) and after outpatient rehabilitation (t2) according to the European Respiratory Society guidelines [24,25].

### 2.4. Ethics Approval

The Ethics Committee of the Medical University of Vienna approved the study protocol on 13 June 2022 (EC Nr: 1161/2022) in accordance with the current version of the Declaration of Helsinki. 

### 2.5. Outcome Measurement

We evaluated anthropometric data, including age, gender, height, weight, body mass index (BMI), and smoking behavior, as well as the COPD disease severity according to the GOLD (Global Initiative for Chronic Obstructive Lung Disease) classification. 

Exercise tolerance was investigated using the 6MWT [18,19], which estimates the cardiovascular and pulmonary performance of a patient below the anaerobic threshold. It measures the distance that a patient can walk as quickly as possible over six minutes on level ground. Walking aids and breaks are allowed. The 6MWT was performed once at baseline (t1) and after outpatient rehabilitation (t2) according to the European Respiratory Society guidelines [22]. All data were collected during routine testing and stored in a password-protected, in-house database. 

### 2.6. Statistical Analysis

We used SPSS for Windows (version 27) for data analysis. For each patient, 6MWT score differences (changes, Δ) between the beginning (t1, pre-test score) and the end (t2, post-test score) of rehabilitation were calculated and presented graphically. *Z*-values and effect sizes for within-subject designs were calculated (Cohen’s *d* and partial Eta-squared, η_p_^2^). Effect sizes were interpreted according to Cohen [26]. For the classification of effect sizes according to Cohen’s d, the following ranges were chosen: very small effect size [VS]: *d_z_* 0.01–< 0.20, small [S]: *d_z_* < 0.5, medium [M]: *d_z_* < 0.8, large [L]: *d_z_* < 1.2, very large [VL]: *d_z_* < 2.0, and huge [H]: *d_z_* ≥ 2.0 [26]. 

**Figure 1:**
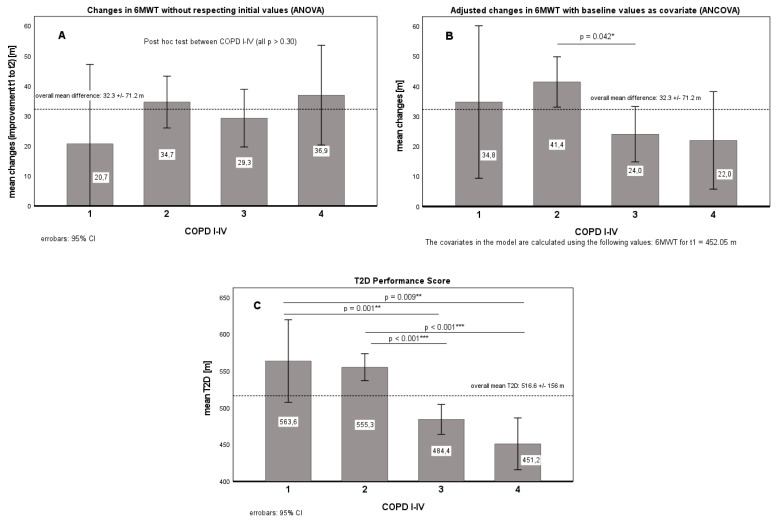
Changes from t1 to t2 in walking distance without (**A**) and with adjustments (**B**,**C**) for baseline values. Figure 1A: no adjustments, COPD group: *p* = 0.636 (part. Eta^2^ = 0.003); Figure 1B: with covariate (6MWT_t1_); covariate: *p* = 0.000 *** (part. Eta^2^ = 0.098), COPD group: *p* = 0.035 * (part. Eta^2^ = 0.015); Figure 1C: T2D, COPD group: *p* = 0.000 *** (part. Eta^2^ = 0.081); Significance level: * *p* < 0.05, ** *p* < 0.01, *** *p* < 0.001.

In addition to multiple paired *t*-tests (exploratory, without alpha adjustments), an ANOVA in a 4 × 2 design (between-factor: COPD disease severity; I–IV x within factor: time; pre, post; resp. changes (D) for ANCOVA from admission (t1) to discharge (t2)) was calculated with each patient’s baseline value (6MWT) at time t1 as covariates (for D; cf. Figure 1). 

#### Performance Score Stratification (T2D)

As patients with poor baseline scores (t1) often appear to respond better to interventions, it is necessary to pool the data correctly and to correct for the tendency for different baseline values to occur. The simple formula T2D = t2 + (t2 − t1) reflects the individual performance and considers the functional status and the change relative to baseline [15,16,17]. This approach has the advantage that groups (between-subject factors) with different baseline distributions can be compared in a pre–post (within-subject) design without fear of mathematical coupling or regression effects, as can be the case with ANCOVAs [27]. 

**Figure 2:**
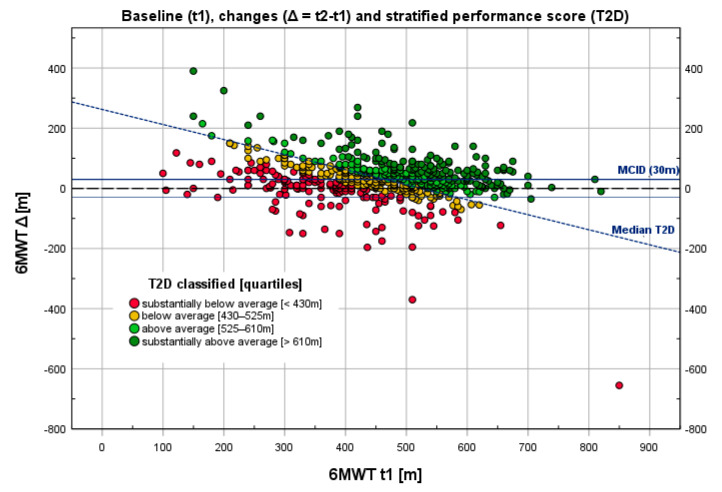
Relationship between changes in 6MWT (Δ: t2 − t1) relative to baseline (t1) and stratified performance scores (T2D). A total of 575 patients with COPD were evaluated through the six-minute walking test (6MWT) before (t1) and after (t2) outpatient pulmonary rehabilitation. There is a negative correlation (*r* = −0.30, *p* < 0.001 ***) between the baseline (abscissa) and changes (ordinate).

Participants were stratified based on the quartiles of T2D with respect to the entire group, with the quartiles being assigned values of substantially below average, below average, above average, and substantially above average (Figure 2). A predetermined MCID of 30 m as defined by international consensus [22] was used for this analysis. 

## 3. Results

### 3.1. Anthropometric Data

A total of 884 patients with COPD underwent outpatient pulmonary rehabilitation, of whom 575 fulfilled the inclusion criteria for this retrospective analysis (Table 1). A proportion of 38.3% of the included patients were female; 4.9% were categorized as GOLD I, 45.7% as GOLD II, 37.0% as GOLD III, and 12.3% as GOLD IV. The average age of the patients was 61.2 ± 9.0 years. The mean body mass index (BMI) was 26.8 ± 5.5 kg/m^2^. A proportion of 28.3% of included were smoking at the time of data collection, and the others (69.7%) were either non-smokers or had stopped smoking before participating in the rehabilitation program. Data on smoking behavior were missing for 1.9% of patients. 

### 3.2. Endurance Performance (6MWT)

In total, 575 COPD patients accomplished a treatment intensity of at least 75% of treatment units approved by the insurance carrier and were therefore considered for data analysis of the effect of pulmonary outpatient rehabilitation on improvements in 6MWT performance (Table 2). The mean change in walking distance after treatment compared to baseline was 32.3 ± 71.2 m. COPD patients classified as GOLD I exhibited a mean improvement of 20.7 ± 42.9 m, GOLD II patients improved by 34.7 ± 76.1 m, GOLD III by 29.3 ± 66.4 m, and GOLD IV by 36.9 ± 75.3 m throughout the program. More than half of the patients in the subgroups reached the minimal clinical important difference (MCID) of 30 m (COPD I–IV: 54.1%; COPD I: 50%, II: 55.1%, III: 51.6%, IV: 59.2%; *p* = 0.671). All pre–post changes were statistically significant (*p* < 0.001), although with small effect sizes (Table 2). 

As shown in Table 2, patients with a higher severity of COPD generally exhibited significantly worse endurance performance (between-group effect: *p* < 0.001 ***, part. Eta^2^ = 0.132; post hoc test_LSD_: COPD I vs. III, IV; COPD II vs. III, IV; COPD IV vs. I–III, all *p* < 0.01 **; COPD I vs. II, n.s.) but a similar increase at the end of outpatient pulmonary rehabilitation (*p* = 0.636, see Table 1 and Figure 1A). Patients who achieved a minimal clinically significant difference of 30 m (MCID) achieved comparable improvements in 6MWT, regardless of GOLD classification (COPD I-IV: 54.1%; COPD I: 50%, II: 55.1%, III: 51.6%, IV: 59.2%; *p* = 0.671 (chi-square, *df* = 3), although there was generally a negative correlation (*r* = −0.30, *p* < 0.001 ***; cf. Figure 2) between baseline (t1) and changes (Δ: t2 − t1). 

In contrast, with respect to the individual baseline results (t1) as covariate (Figure 1B), COPD II patients exhibited, on average, a 17.4 m greater improvement in 6MWT from t1 to t2 relative patients with COPD III (CI: 0.4–34.4 m, *p* = 0.042 *). Accordingly, the classification based on MCID also differed (COPD I–IV: 53.7%; COPD I: 57.1%, II: 63.1%, III: 43.7%, IV: 47.9%; *p* = 0.000 *** (chi-square, *df* = 3)). The use of T2D showed further differentiation (Figure 1C). Patients with COPD I and COPD II (CI: 539–574 m) showed, on average, better T2D performance (80 +/− 13 m) than patients with COPD III and COPD IV (CI: 459–494 m). Classification based on above-average T2D performers (median split) yielded the following frequencies: COPD I–IV: 51.0%; COPD I: 67.9%, II: 62.0%, III: 39.0%, IV: 23.9%; *p* = 0.000 *** (chi-square, *df* = 3).

Figure 2 demonstrates the relationship between the 6MWT at baseline (t1) and the corresponding change in 6MWT (t2 − t1). The values are stratified according to the quartiles of the T2D, with assigned values ranging from “substantially below average” to “substantially above average”, as indicated by different colors. The range of the absolute values of T2D in this sample are stated in the legend. 

The discrepancy between the classification participants using the MCID and the T2D, as shown in Figure 2, is quantified in Table 3. Goodman and Kruskal’s gamma showed a strong correlation between the MCID and T2D (gamma = 0.658, *p* < 0.001 ***), whereas McNemar’s test showed significant discordance between the two (*p* = 0.037 *). However, when using adjusted values (adjusted for baseline value), as shown in Table 3 (brackets), the correlation of MCID with T2D was nearly perfect (gamma = 0.923, *p* < 0.001 ***), with McNemar’s test showing an even greater discordance. 

The combination of the binary classifiers of MCID and T2D (Table 3) provides an objective method for estimating cases with ceiling effects (13.2%) and floor effects (18.3%), which can be reduced to 6.3% (false negative) and 11.0% (false positive), respectively, by covariance-adjusted values for MCID.

## 4. Discussion

In this retrospective cohort study, we analyzed the changes observed in exercise capacity during pulmonary outpatient rehabilitation and applied a recently introduced integrated performance measure, the “performance score” (T2D), in patients with COPD for the first time.

Previously, patients were considered to perform well in the 6MWT if changes in walking distance achieved a certain threshold at which an observed difference in walking distance was perceived as important for the patient. This threshold is referred to as the minimal clinically important difference (MCID) [20]. A review of the literature revealed some controversy regarding the minimal important difference of improvements in the 6MWT in COPD patients [8,20,28,29,30] and across multiple patient groups [20,31]. Holland et al. [22] presented an official technical standard of the European Respiratory Society/American Thoracic Society, noting an MCID of 30 m, which is the value we used in our analysis. The mean change in walking distance after treatment compared to baseline across the 575 COPD patients of all stages was 32.3 m; thus, more than half of the patients reached the MCID. Rehabilitation resulted in statistically significant improvements in the 6MWT walking distance (*p* < 0.001), with a small effect size (Cohen’s *d* = 0.45). These findings are in accordance with multiple studies, confirming that pulmonary rehabilitation of varying duration, frequency, intensity, and type, leads to improvements in walking distance in patients with COPD [8,32,33,34].

However, the approach of evaluating absolute changes in walking distance disregards the fact that varying baseline values play a role in assessing performance. As patients with poorer baseline scores (t1) often appear to respond better to interventions, differences between the start (t1) and end (t2) of a rehabilitation program may not serve as valid indicators of what patients and clinicians report. These changes depend on the patient’s baseline status (Figure 1A,B). Thus, an improvement of 30 m represents a 10% increase for a person with a baseline walking distance of 300 m but only a 5% improvement for a person who walks 600 m at baseline. Different individual baseline values must always be taken into account, as poorer outcome measurements at the beginning of rehabilitation are accompanied by greater potential for improvement [35]. Therefore, it is necessary to pool the data correctly and to correct for the tendency for different baseline values to occur. Stratified outcome scores for evidence-based determinants of health status are essential for healthcare delivery because the baseline medical condition has the strongest moderating effect on outcome [36]. The “performance score” (T2D) reflects the fact that patients with a short baseline walking distance have to improve more than individuals with a long baseline walking distance to perform well. The line separating above-average and below-average performers (Figure 2) runs from the upper left to the lower right area of the graph, indicating that equal performance requires more considerable improvements in patients with low baseline values and less considerable improvements in those with high baseline values. Thus, even a patient with no improvement between the baseline and post-treatment 6MWT (Δ: t2 − t1= 0) can perform well if he/she achieved a high value (in this case, more than 525 m) at baseline.

The “performance score” assesses individual scores within a patient, considering the patient’s health status at baseline (t1) and after rehabilitation (t2), as well as the patient’s progress made during the rehabilitation process (changes; Δ). The individual patient’s performance scores can be classified from substantially below to substantially above average. As illustrated in Figure 2, the score could potentially be used to differentiate between responders and non-responders in the rehabilitation process in a simple and user-friendly way. Therefore, this appears to be a promising alternative approach to conventional ways of evaluating patient performance and presenting rehabilitation outcomes.

As shown in Figure 1A, simple values of changes in 6MWT seem the greatest in the patient group starting rehabilitation with the lowest values. When correcting for baseline values, a more realistic picture of the relative changes seems to appear (Figure 1B). However, this tendency is only revealed in graphic illustration of the T2D (Figure 1C), which seems to represent the expected deterioration in 6MWT across GOLD stages while also accounting for changes over time.

The discrepancies between the MCID and T2D, as shown in Figure 2 and Table 3, seem to suggest that the performance score is more accurate at assessing performance than the MCID. McNemar’s test, in particular, suggests that T2D performs better at identifying below-average performers who have managed to reach the MCID due to a low baseline value. However, as suggested by most values, as the MCID is distribution-based, in the absence of an anchor-based gold standard, the effects of the different methods cannot be ascertained with absolute certainty. The use of the T2D seemed to have an effect similar to adjusting the values of Δ by factoring in baseline 6MWT values (Figure 1A,B, Table 3). The MCID correlated nearly perfectly with T2D after individual values were adjusted for baseline 6MWT values (gamma = 0.923, *p* < 0.001). Most importantly, the combination of MCID and T2D provides an objective method for estimating false-positive and false-negative outcomes. Due to its simplicity, T2D may prove to be an intuitive and user-friendly tool in the future.

### 4.1. Limitations

For ethical, practical, and economic reasons, a randomized controlled design could not be applied to this study.

Another limitation is that the application of the T2D and the benefits of the easy-to-apply classification of rehabilitation success assume knowledge of default standard values for a certain population. Thus, the classification into responders versus non-responders as demonstrated in Figure 2 is only applicable for this sample of COPD patients undergoing outpatient rehabilitation evaluating the 6MWT outcome.

Only 575 of 884 patients accomplished the prescribed therapy as approved by the insurance carrier, with available values for both 6MWTs, and could therefore be considered for data analysis. This possibly influenced the study results. Despite the standardization and the excellent reliability of the primary outcome (6MWT), group-specific variability of the result, e.g., due to the influence of learning effects, cannot be excluded.

Furthermore, the performance score was calculated based on the entire sample across GOLD stages and phases of rehabilitation (phase II and phase III), which may affect the applicability of the presented reference values for the performance score to patients with differing GOLD stages or undergoing pulmonary rehabilitation of different phases and over different periods.

### 4.2. Implications for Future Research

Future studies should aim to collect a sufficient set of data to generate standard values for other patient groups, outcome measures, and interventions and explore the acceptance of this new measure by health care professionals. In addition, future research should explore the utility of the performance score (T2D) in predicting long-term outcomes in COPD.

## 5. Conclusions

In this study, the recently introduced performance score (T2D) was applied for the first time for outcome evaluation of COPD patients undergoing outpatient pulmonary rehabilitation. Using a distribution-based approach, the score considers the patient’s current status, in addition to changes over time. Thus, this single parameter appears suitable to effectively differentiate responders from non-responders in the rehabilitation process in a simple and user-friendly way, representing a promising alternative approach to commonly used methods of interpreting outcome changes in COPD.

## Figures and Tables

**Table 1:** Anthropometric data, smoking behavior, and COPD GOLD classification of included patients.

	*N* (%)	Sex		Age		BMI		Smoker	
		Female	Male	Mean	SD	Mean	SD	No	Yes
COPD all stages	575 (100)	222	352	61.2	9.0	26.8	5.5	401	163
COPD I	28 (4.9)	10	18	63.0	10.5	26.6	6.2	23	5
COPD II	263 (45.7)	107	155	61.1	9.3	27.9	5.2	186	73
COPD III	213 (37.0)	82	131	61.9	8.6	26.1	5.2	142	66
COPD IV	71 (12.3)	23	48	59.4	8.0	24.4	6.2	50	19

Legend: %: percent; SD: standard deviation; sex and age one missing value; missing for BMI (5 cases); missing date for smokers (11 cases) < 2%.

**Table 2:** The effect of pulmonary rehabilitation on the six-minute walking test (6MWT).

Group	*n*	6MWT t1 [m]	6MWT t2 [m]	6MWT Δ [m]	Cohen’s d
COPD I–IV	575	452.1 ± 118.6	484.3 ±119.0	32.3 ± 71.2 ***	−0.45 [S]
COPD I	28	522.2 ± 93.2	542.9 ± 100.0	20.7 ± 42.9 ***	−0.48 [S]
COPD II	263	486.0 ± 108.9	520.6 ± 108.2	34.7 ± 76.1 ***	−0.45 [S]
COPD III	213	425.8 ± 111.2	455.1 ± 116.2	29.3 ± 66.4 ***	−0.44 [S]
COPD IV	71	377.3 ± 126.9	414.3 ± 116.8	36.9 ± 75.3 ***	−0.49 [S]

Legend: m: meter; t1: before rehabilitation; t2: after rehabilitation; Δ: t2 − t1 (change, D); *** *p* < 0.001 pre to post; [S]: small effect size.

**Table 3:** Assessment of changes in endurance performance with MCID classification vs. T2D classification.

Contingency Table of Binary Classifiers		T2D (Median Split)		∑
(Absolute Frequencies)		Below Average (<MED)	Above Average (>MED)	
MCID (MCID adj.)	<30m	188 (230) ^a^	76 (36) ^b^	264 (266)
	>30m	105 (63) ^c^	206 (246) ^d^	311 (309)
Total (∑)		293	282	575
MCID vs. T2D: Gamma = 0.658 ***, Kappa = 0.371 ***, McNemar: *p* = 0.037 * (MCID adjusted vs. T2D: Gamma = 0.923 ***, Kappa = 0.656 ***, McNemar: *p* = 0.009 **) Significance level: * *p* < 0.05, ** *p* < 0.01, *** *p* < 0.001				

^a^ Non-responder (no improvement): 32.7% (40.0%); ^b^ ceiling effect due to already good 6MWT performance at baseline (MCID can be evaluated here as false negative): 13.2% (6.3%); ^c^ floor effect due to poor 6MWT performance at t1 that improved at t2 in a way that is not relevant to health (MCID false positive): 18.3% (11.0%); ^d^ responder (clinically important improvement): 35.8% (42.8%); cf. Figure 2 (MCID vs. T2D).

## Data Availability

The raw data can be provided upon reasonable request. Requests for access to anonymized datasets should be directed to the corresponding author.
